# Conceptualising effective symptom management in palliative care: a novel model derived from qualitative data

**DOI:** 10.1186/s12904-022-00904-9

**Published:** 2022-02-04

**Authors:** Emma J. Chapman, Simon Pini, Zoe Edwards, Yousuf Elmokhallalati, Fliss E. M. Murtagh, Michael I. Bennett

**Affiliations:** 1 Academic Unit of Palliative Care, Leeds Institute of Health Sciences, Worsley Building, Clarendon Way, Leeds, LS2 9NL UK; 2 Wolfson Palliative Care Research Centre, Hull York Medical School, University of Hull, Hull, HU6 7RX UK

**Keywords:** Pain, Dyspnoea, Psychological distress, Fatigue, Palliative medicine, Hospice, Neoplasms

## Abstract

**Background:**

Pain, breathlessness and fatigue are some of the most challenging symptoms to manage in patients with advanced disease. Specialist palliative care leads to better symptom management, but factors contributing to successful symptom management in this context have not been explored. Our aim was to understand what facilitates effective symptom management in specialist palliative care within UK hospices and investigate what barriers are experienced.

**Methods:**

This was a grounded theory study using qualitative semi-structured focus groups and interviews. Participants were recruited from multidisciplinary specialist palliative care teams (doctors, nurses, healthcare assistants, physiotherapists, occupational therapists, complementary therapists, social workers and chaplains) working in inpatient, outpatient and community services provided by five hospices in the United Kingdom.

**Results:**

We present a novel qualitative data-derived model of effective symptom management in specialist palliative care. We describe a co-ordinated, multi-faceted, sequential approach involving a process of engagement, partnership, decision-making, and delivery. Interventions to manage symptoms are less effective in psychologically distressed patients. Our data highlights that families of patients have a key role in determining effectiveness of symptom management interventions A holistic approach by a co-ordinated, multi-disciplinary team, including support to recognise and minimise psychological distress might facilitate more effective symptom management. Barriers to symptom management include team discordance and lack of understanding about symptom management by patient and families.

**Conclusions:**

Shared decision-making between patients and professionals and co-ordination of care by a multi-disciplinary team are key components of effective symptom management. Actions to address psychological distress and evaluate the understanding and expectations of patients and their families would enable more effective symptom management. A more effective multi-disciplinary approach would be facilitated by discussion within teams about role competencies and boundaries.

**Supplementary Information:**

The online version contains supplementary material available at 10.1186/s12904-022-00904-9.

## Background

In the United Kingdom (UK), palliative care is provided free of charge for people with progressive and advanced disease. Care is delivered in hospitals and hospice-based out-patient clinics, home care teams or on in-patient units. A key aim of palliative care is to optimize management of troubling symptoms. A symptom can be defined as a ‘subjective experience reflecting changes in biopsychosocial functioning, sensations, or cognition of an individual’ [1]. The theoretical conceptual framework of comfort in nursing care developed by Kolcaba [2], defines providing comfort as the ‘immediate experience of being strengthened by having needs for three types of comfort (relief, ease or renewal) met in four contexts of human experience (physical, psychospiritual, environmental and social)’. Effective symptom management aims to provide comfort and has the potential to increase quality of life for patients and families. Support from specialist palliative care is associated with less symptom burden at home [3] or in hospital [4].

Over 20 years ago, the University of California, San Francisco School of Nursing developed a model for symptom management [5]. Their conceptual model was designed to ensure a comprehensive approach to symptom management in clinical practice and was made up from over-lapping interactions of symptom experience, management strategies and outcomes. The model was revised in 2001 to include influence of person, health/illnesses and environment [6] and has been applied in multiple contexts as a theoretical framework [7]. Current practices, challenges and facilitators to delivering effective symptom management in specialist palliative care have not been investigated. We aimed to explore the barriers and facilitators to effective symptom management in UK hospice-based specialist palliative care.

## Methods

Our aim was to understand what facilitates effective symptom management in specialist palliative care and investigate what barriers are experienced.

### Theory

Grounded theory [8] was employed to develop a theoretical understanding of effective management of common symptoms within palliative care. Having been unable to identify an existing theory specific to symptom management in this research population, grounded theory was chosen as an appropriate methodology to allow fresh exploration of the topic, pursuit of emerging areas of interest during data collection, and ultimately the development of new theory. This research is underpinned by a pragmatic epistemology [9, 10]. The study is reported in accordance with Standards for Reporting Qualitative Research (SRQR) recommendations [11].

### Settings and participants

Data was collected between May and November 2019 from five hospices in north of England. Participants were eligible if they worked in a healthcare role within a participating hospice. Hospice staff provided outpatient, inpatient and community care. In keeping with a core principle of grounded theory, theoretical sampling was used to identify participants and further pursue emerging data. Participant roles were classified as doctors, nurses (including healthcare assistants), and allied healthcare professionals (including chaplains, social workers, physiotherapists, occupational therapists and complementary therapists).

### Ethical approval

All methods were carried out in accordance with the principles of the Declaration of Helsinki. Written informed consent was obtained from participants. Ethical approval was granted by University of Leeds research ethics committee (MREC 18-065, 11/3/2019).

### Study procedure

Initially, staff were invited to participate in a role-specific focus group or interview at their usual place of work by their head of service or research nurse on an opportunity sampling basis. Focus groups followed a standard semi-structured approach informed by the interview guide. As new information emerged throughout focus groups, individual interviews were arranged with additional participants of the same staff category to further explore interesting or novel topics. Participants were asked to read study information at least 24 h before meeting and written informed consent was obtained on day of interview/focus group. One potential focus group participant (a student) completed the consent process but then did not participate as it was their first day working in palliative care and they had no experience to reflect upon. No data was collected from this person and their demographic information was not retained. Interviews/focus groups were audio-recorded onto an encrypted, password protected dictaphone and professionally transcribed verbatim. Recordings and transcripts were stored on a secure server at University of Leeds. Data was pseudo-anonymised in that each participant was allocated a study identification number, only research team could link identification number with staff identity.

### Data collection

Semi-structured focus groups or interviews were carried out with 61 participants Fifteen role-specific focus groups (with 2 to 5 participants) were held to identify barriers and facilitators. Focus groups involved nurses (20 participants), doctors (11 participants) and allied healthcare professionals (22 participants). Subsequently, six individual and one shared interviews were held to collect more in-depth data on emerging topics. Interview participants were one nurse, five doctors and two allied healthcare professionals. Eleven participants had a specialised palliative care qualification. Due to the gender imbalance in the palliative care workforce towards females, gender is not reported as this may compromise confidentiality. Demographic information of participants and focus groups and interview composition is detailed in Additional material Tables 1 and 2. Facilitators were University researchers, with experience in palliative care research and qualitative methodology. Quotations from different focus group participants are identified as P1, P2 and interviewer as I1, I2. Our first objective was to capture healthcare professional’s current practices in management of pain, breathlessness and fatigue in patients with advanced disease. Focus groups began with a card sort exercise [12] where participants placed commonly used approaches to symptom management into piles of “Would use” and “Would not use”. Approaches identified on cards were drawn from guidelines, literature and experience within the research team. This exercise aimed to stimulate discussion of symptom management strategies and identify other approaches, which were added to blank cards. Participants were encouraged to discuss examples of when their approach to symptom management had been successful or problematic. A semi-structured topic guide was used (Table 1), to help participants elaborate on their examples, but researchers were guided by participants where possible. Card sort exercise was not utilized with chaplains and social workers as they were not directly responsible for symptom management. Having completed this introductory exercise we progressed to asking staff to reflect upon what impacted on treatment decisions and success of their approach.

**Table 1:** Topic guide for healthcare professional focus groups

Section	General Question	Prompt items
**Introduction**	Introduce Research Looking at how to make it easier to manage symptoms in advanced cancer patients	• Introduce self • Explain confidentiality, length of interview/group, nature of discussion • What we are going to cover • Any questions? • Obtain consent • Start recording • Participants invited to introduce themselves
**Assessing and managing challenging symptoms**	How do you manage Pain Breathlessness Fatigue	• Can you talk me through your approach to managing each of these symptoms? (Card sort exercise). • How do you decide what to do? (guidelines?) • How consistent is the approach to managing these symptoms in your workplace? • How often are you completely satisfied that the patients symptoms are optimally controlled? • Most/least confident managing? Which is the most severe and which has the biggest impact on quality of life
**Example of good experience and possible facilitators**	Can you think of a situation when you were able to manage one or more of these symptoms well	• How would you describe the experience? How did this make you/patient/carers feel • Why was it that in this case you were able to manage the symptom well? What/who helped you?
**Example of poor experience and possible barriers**	Can you think of a situation when you were not able to manage one or more of these symptoms so well	• How would you describe the experience? How did this make you/patient/carers feel • Why do you think that in this case it was not possible to manage the symptom well? • What made it so difficult? • Who else can you ask for help if the symptom cannot be controlled? • Who might you work with? Other in same role/nurse/doctor/pharmacist/complementary therapist/ physiotherapist? • What do you think could be done (in an ideal world) to make management of these symptoms more successful more often
**Closing**	Thank you for your participation	

### Analysis

Grounded theory has developed and diverged since it’s foundation in work of Glaser and Strauss [8]. Despite the variety of approaches, four core elements are present in all grounded theory work: theory grounded in collected data, explaining context-related processes, pursuing theory through engagement with data, and using theoretical sampling [13]. Using this approach, we employed an iterative process, collecting and analysing data concurrently. Authors met regularly to discuss and refine emerging concepts, integrate newly collected data and identify areas requiring further targeted exploration. A thematic framework was tentatively developed to facilitate exploration of transcripts (Table 2), this remained flexible and adaptable throughout analysis and writing-up process. See Additional material Table 3 for illustrative extracts.

**Table 2:** Thematic framework

**1. What influences healthcare professional’s choice of approach?**	
1.1 Guidelines and Evidence	
1.2 Experience	
1.3 Training	
1.4 Role definition and boundaries	
1.5 Multidisciplinary team decision making	
1.6 Availability of services/staff	
1.7 Clinician–Patient relationship/rapport	
1.7.1 Patient preferences	
1.7.2 Patient characteristics (including reversible causes)	
1.8 Quality of life versus treatment need	
1.9 Staff time/burden	
**2. What do healthcare professionals see as important factors in supporting delivery of effective care?**	
2.1 Psychological support	
2.1.1 Formal	
2.1.2 Informal	
2.1.3 For staff	
2.2 Appropriate understanding, expectations, acceptance and goals	
2.2.1 Patients	
2.2.2. Healthcare professionals Family/carers/friends	
2.3 Professional, service and referral factors	
2.3.1 Continuity of care	
2.3.2 Multidisciplinary team working	
2.3.3 Palliative care philosophy and culture	
2.3.4 Physical environment and facilities	
2.3.5 Referral process and delays	

**Fig. 1:**
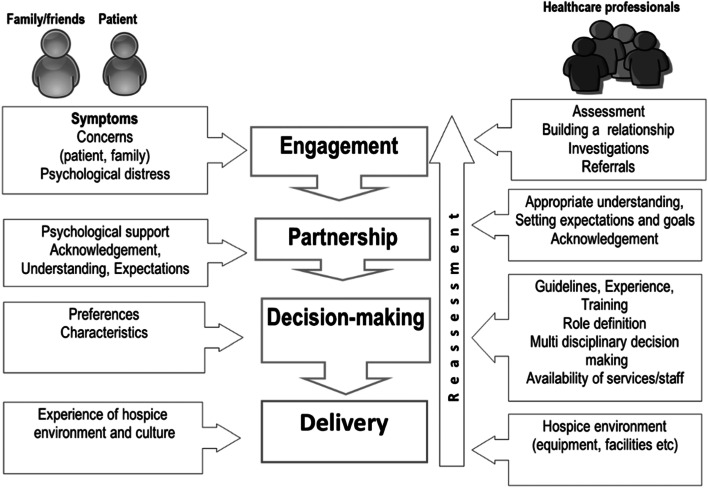
Conceptual Model of effective symptom management

Conceptual mapping was conducted alongside developing the thematic framework to allow authors to explore how emerging themes interacted and overlapped to facilitate novel theoretical directions to inform our novel conceptual model (Fig. 1).

## Results

We depict symptom management in palliative care as a logical sequence of interacting domains, each of which contributes to an overall effective response. Each domain of engagement, partnership, decision-making, and delivery is influenced by a combination of patient, family, professional and service provision factors. Pragmatic suggestions of how these might be targeted to maximise symptom management success are listed in Table 3.

**Table 3:** Suggested strategies for more effective symptom management

Supporting patient Engagement	Simple initial screening to identify potential psychological distress which may be a barrier to engagement with symptom management
	Act to minimise psychological morbidity through low level informal support or referral for specialist care where necessary
Partnership	Discuss patient’s understanding, expectations and goals
	Empower family members with understanding of symptoms and their management
Decision-making	Agree shared plan of action with patient based upon patient preferences, treatment guidelines, and multi-disciplinary team experience
Delivery	Deliver and co-ordinate a multi-disciplinary approach based upon good understanding of other specialities and roles

### Engagement

Engaging patients in a symptom management strategy was a gradual process that enabled and empowered patients to choose which approach to take.

*P2: it does take time and I think of it as just planting seeds of information that grow throughout the process of you working with them (Occupational therapist)*

*P4: There’s perhaps a lack of understanding on what our interventions involve and how a lot of them do take weeks of working with someone directly. (Physiotherapist)*

*P1: I just tend to give people all the information I had a lady just like that a couple of days ago and erm I’ve just given her information about things she could do…and then give her the choice of whether she wishes to do that or not and if she doesn’t, she knows where I am for the future…you’re not going to make a switch overnight with these people.(Occupational therapist)*

Having time to get to know patients was necessary to build up a therapeutic relationship, this facilitated development of a tailored approach**.**

*my first session is usually about an hour talking to that patient … really kind of again building up that therapeutic relationship and getting to know that individual and that holistic assessment. (Occupational therapist).*

All groups of healthcare professionals said interventions to manage symptoms were less effective in psychologically distressed patients. This topic was raised in every focus group or interview. Allied healthcare professionals were key in providing informal psychological support and recognised a need to address anxiety and distress before attempting to manage symptoms.

*Anxious people is a real challenge for me because you can reason with them, but they are just so anxious, it’s as if they have an answer for everything, as in you would go, “Okay, well let’s try and solve this problem,” but then that would make another problem…they are so anxious that they can’t take the strategies or they don’t want the equipment and things like that, I find that quite challenging. (Senior Occupational Therapist)*

Staff reported patients were fearful of opioid drugs due to risk of addiction and negative portrayal in the media.

*on the news has been the whole debate about opiates and people becoming addicted and I think personally, more so over the last couple of years, people are much more reluctant to take things. (Staff nurse, out patients unit)*

This could be a barrier to their use and a reason for patients being reluctant to engage with treatment.

*they recommended that she starts on low dose morphine for her pain, for her breathlessness and she said I’m not sure about that and I said why are you, she said I don’t want to become addicted to it (Staff nurse, outpatients unit).*

The concept of multifactorial pain with an emotional component was frequently discussed.

*I think complex pain is multifactorial, so it’s not necessarily just the physical pain. So, there’s a couple of patients…who were really, really struggling emotionally as well, but I think they’re really, really challenging because there’s not really a tablet or a painkiller to treat that (Doctor, Registrar in Palliative Medicine, inpatient unit)*

This doctor recognised that without identifying and addressing the patient’s psychological distress, medical intervention they could provide, for physical symptoms such as pain, were less effective.

*If they hadn’t had the psychological burden dealt with, then any intervention that you use is less effective. (Speciality doctor palliative care).*

Having described how psychological pain made physical pain so much harder to manage, this social worker described a case where identifying and targeting a source of psychological distress relieved physical symptoms.

*She had a son who was in prison who she’d not seen for many months and her focus was to see her son who had now been moved to a secure mental health unit. We facilitated that happening and that brought her a lot of symptomatic relief actually. (Social worker)*

Other staff reflected whether even in specialist palliative care, psychological issues were not addressed as well as they could be.

*So she was a very anxious lady and I think that there was a psychological component to her pain. …I don’t know if even here if we’re guilty of ignoring that. Not ignoring it but not, not taking steps to deal with that and we try, the counsellors see people but I wonder if a psychologist would be helpful. (Doctor, GP trainee)*

Despite staff acknowledging the huge role anxiety and psychological stress has in symptom experience, their consultations focused on formal descriptions of pain, and options for treatment of physical symptoms and medication. Sometimes it was patients and their previous experience of consultations that lead this style of conversation and perhaps staff did not intervene to gain a better understanding of symptom experience.

*we have a learned behaviour of talking to doctors and we tell them the information that we think that the doctors are interested in so every time you see a doctor and you talk about pain they ask you where is it? When does it come? Where does it radiate? What’s it like? You know the Socrates stuff… so in the hospice they don’t necessarily talk about how badly it affects them and what it means to them. (Doctor, GP trainee)*

Staff were highly motivated to improve their knowledge of psychology and this was often done in their own time.

*we’re trying to upskill on that …but we’re not erm you know none of us are kind of CBT (Cognitive Behavioural Therapy) practitioners or anything like that or, and we haven’t had, we’re learning about ACT (Acceptance and Commitment Therapy) and that but we’re not trained in that so using principles of those things in what we’re doing with people so yeah (Physiotherapist).*

### Partnership- within the multi-disciplinary team

Being treated by a team with the ability to call upon each other’s strengths contributed to effective symptom management.

*I think the strengths of the unit are that we’ve got a very diverse team; we’ve got good allied health professional departments that are really, really experienced and very friendly and open to suggestions, and we’ve got a very non-hierarchical team. (Doctor, Registrar in Palliative Medicine)*

Some patients were treated by staff who lacked knowledge of what other departments or staff could provide. One nurse did not really know what was available from complementary therapy, their use of “as a nurse” implies it is not their role to know.

*I know as a nurse I don’t ask, I haven’t asked so maybe that’s something for me to look at, I don’t’ know what they offer (Nurse, transfer of care, inpatient unit)*

Patients were routinely referred to physiotherapists and occupational therapists at the hospice but when asked, the referring doctor admitted that they had no idea what they would provide.

P*: in terms of fatigue management, we’d use again Physios (Physiotherapists) and OTs (Occupation therapists); I think they’re really useful in these situations, and they do fatigue management.*

*I: So, what is it you think they provide?*

*P: To be honest, I’ve no idea. I’ve no idea; I should probably go and sit in on what they do. (Doctor, Registrar)*

Staff felt that they were aware of their own limitations and only spoke with patients and carers about issues they were able to. Where and how these unspoken boundaries are set was unclear and occasionally caused conflict. For example, one doctor, despite understanding the importance, felt they couldn’t suggest simple measures to alleviate breathlessness to the patient as this was not within their remit and was nurses’ responsibility.

*Positioning’s a big deal…, it’s something that we use but it’s not in my remit if that makes sense so they, you know it’s not really my responsibility to say sit up, it’s the nurses who are going in constantly to see these patients repositioning them and sitting them…you sometimes feel a little bit like you’re teaching you’re telling somebody how to do their job and sometimes that’s a bit of a difficult position to be in. There’s no formal line about whose responsibility it is but it just sort of falls that way (Doctor, GP trainee, interview)*

A high turnover of staff had a negative effect on training and relationship building with other team members. There was a necessity for a continuous cycle of training and skill sharing.

*there’s often a high turnover of staff so you think you’ve kind of done lots of education sessions and training sessions with people which we have started to do with other hospice staff and they leave and then there’s new starters and it needs to be ongoing. (Physiotherapist)*

### Partnership – interactions between patient and professionals

The simple step of giving patients acknowledgement of their symptom and distress it was causing helped to build up a supportive therapeutic partnership. This was particularly valuable in the management of fatigue.

*the big one like you said is acknowledgement ‘cos some people at least then I think a weight’s been lifted when they know that this is a normal part of their, their disease and we can’t necessarily cure it but we could hopefully improve it but they need to be involved with that. There isn’t a magic drug. (Nurse, inpatient unit)*

Managing expectations with honesty was important and enabled focus on and finding value in what was achievable rather than setting unrealistic goals.

*one of the most important parts of our job is about trying to manage someone’s expectations setting goals for what is realistic, what isn’t realistic because actually when you accept what’s not achievable then actually finding quality and value in what is achievable is becoming more possible really. (Doctor, Associate Specialist, inpatient unit).*

A key action was to clarify whether expectations were directed towards affecting level of pain or whether improvement in functional capacity could be achieved.

*part of the self-help is that issue of OK what are we aiming for? Are we aiming for improved function and accepting a degree of pain or are we aiming for complete pain relief and more often than not it’s improved function (Doctor, Consultant in Palliative Medicine)*

There was a need for clear communication of complex information to patients and family about cause and treatment of symptoms.

*I’ve explained this to lots of patients now how adding lots of different drugs is going to be more likely to help their pain than going to a really high dose of just 1 or 2 drugs you know covering lots of receptors and how it’s complicated (Consultant in Palliative Medicine, inpatient unit)*

When patients lacked understanding about pain it contributed to a worse symptom experience. Explaining causes of pain and its variable nature in itself could improve symptoms.

*Certainly it’s funny how sometimes you’ll improve somebody’s pain without having to do much of the kind of stuff just a bit of explanation and discussion around what’s causing it. (Consultant in Palliative Medicine, inpatient unit)*

### Partnership – role of families

The impact of families (by which we mean people important to the patient) as an integral part of successful symptom management was a common theme.

*I think family and carers have a real crucial role in being the someone, just having that calm, reassuring voice and trying to go through getting in the right position to manage breathlessness, trying to do the breathing techniques. Because often when the patient is so breathless and they’re not thinking, they won’t be able to do the relaxation techniques or the breathless techniques. But if there’s someone there who’s not feeling breathless, they can go through it with them. (Registrar interview)*

Helping family to engage with symptom management strategies could help with adherence to and delivery of a symptom management plan.

*carers and family and friends are key to it because they can say, “Yes, yes, yes,” to me for an hour and say they are going to do it and then you go away and they are like, “I’m not going to do it”. So having them on side. (Senior Occupational therapist)*

Staff acknowledged it was challenging to help a family member manage their symptoms, yet very easy to make it worse.

*it’s very difficult to help with the pain like for a family member to help the situation is very difficult but again for them to make it worse is very easy. (GP trainee)*

Family distress made the patient’s experience worse as it was a barrier to family providing effective support.

*they work their family member up because they’re distressed so they sort of play off each other’s stress and the last thing you want when you’re breathless is to get more stressed and worked up …we’ve had a patient recently who’s got end stage COPD (chronic obstructive pulmonary disease) and she, when some of her family members were there her PRN (medication Pro re nata, as required) was sky high (Doctor, GP trainee)*

Negative reinforcement about pain could result in a need for additional pain relief when family were visiting.

*P4: the family can be constantly drilling that into their head and then they think that they are in pain or they’ll say oh so and so‘s in pain and then they might be, they just want to sort of like keep the relatives happy so they go along with it really (Healthcare assistant, inpatient unit)*

*P3: It is definitely with some patients as soon as the visitors arrive then they need the pain relief increasing*

*P3: Families are a massive barrier. We’ve had some really tricky families at the moment and I know that’s not a physical thing that we can particularly change but I think actually educating people (Nurse inpatient unit)*

Families could have a poor understanding about what was going on with their loved one. Use of a syringe driver was perceived to be associated with imminent death.

*there’s a lot of fear isn’t there for erm the syringe driver ‘cos as soon as the syringe driver’s started, in a lot of relatives that’s it their loved ones are going to be you know dead within 24 hours…and it’s communication isn’t it and you know explaining at each level why you’re putting this up and what it’s for. (Nurse, inpatient unit)*

Staff felt some families found it challenging to understand their loved one’s fatigue.

*we have families who think that it’s, this tiredness is just nonsense and they just need to wheel him round the garden 4 times and give him some big food that they’ve read about…and we can’t help them to understand. They are then worried it’s drugs that we’re giving them so we go through all the drugs and erm sometimes stop drugs to demonstrate it isn’t the drug and it doesn’t make any difference erm so we do sometimes have challenging families who are a bit frustrated I think really with what they’re seeing. (Doctor, Consultant)*

When specifically asked about impact of family, this doctor talked about how they would like families to have better understanding of treatment strategies and symptom management. There was a dual motivation for this; to make their own job easier, so family could “not be constantly asking questions” but also for benefit of the family’s peace of mind.

*I would like them to understand enough that they can get on with it and be OK with it, not be constantly asking questions... But there’s another side where I obviously want them to be understanding enough that they’re not distressed and not worried about what’s happening… their family members are dying from their illnesses and it’s a trying time so you just hope that they get some peace in knowing we’re doing everything that we can to stop the symptoms. (Doctor, GP trainee)*

### Decision-making

Decisions on how to treat a symptom were based upon patient preferences and characteristics, guidelines, training, experience and multi-disciplinary decision making.

*we have twice a week the clinical review meetings..where all of the professionals involved in patient care come together and review every single case one by one (Chaplain)*

Pharmacological and non-pharmacological approaches in alignment with current guidelines were used (Additional material Table 4). Others with less supporting evidence such as transcutaneous nerve stimulation were also offered.

Most patients experienced fatigue but staff felt there was not a good evidence-base to support the best way to manage the symptom and found it challenging to know what to recommend..

*I think there’s no doubt that fatigue is one of the things that we struggle to manage, struggle to have strategies to deal with… It feels like you need the fatigue equivalent of a dietician, a “fatigueatician”(Palliative Care registrar)*

### Delivery

The hospice environment itself was reported to relieve symptoms. “Small” differences compared to hospital such as friendly welcome, homely surroundings and being informed about what was happening contributed to improved symptom experience before any medical interventions.

*the thing that I, that surprised me the most that makes the most difference is being in the hospice compared to the hospital…as soon as they’ve come through the door their family’s gone, it’s completely different, they’re much better. (Doctor, GP trainee)*

In contrast, occasionally, sending the patient home was in their best interest.

*it seems really counter intuitive but sometimes the hospice environment is not the right place to be erm and actually being at home might be a different situation (Doctor, Registrar in Palliative Medicine)*

Formal psychological support enabled patients to develop coping strategies to help with self- management and delay or reduce need for medication and staff intervention.

*the moment he was in pain he’d be buzzing for medication and what the psychologist there was able to do was give him a list of different things to try not just buzzing straight away… so instead of thinking immediately the only thing that will solve this is a drug, actually I’ve got this list of things that I can try (Staff nurse, inpatient unit)*

Staff saw a widespread need for psychological support, yet patients who “don’t have time on their side” had to join long waiting lists for formal support delivered by counsellors, psychologists or psychiatrists. Psychological support was described as a “luxury” and something with which hospices could “achieve a great deal more”

*its hugely important that we do have psychology input which is a big issue and something else we’ve also raised to say that a psychologist could really, really help in these sort of situations, probably a lot more than drugs (Doctor, Consultant, inpatient unit)*

Family support teams (social workers, chaplains) provided informal psychological support in complex cases although doctors sometimes felt they were reluctant to intervene as they felt symptoms were “a medical problem”.

*you maybe sometimes get the impression the social workers feel a little bit like, “Oh, this is a medical problem.” Because you hear them when they’re talking about patients, they go, “Oh, I’m not a medic, I’m not a doctor, I don’t know how to say these words, I don’t know what these words mean.” I don’t know if that plays a role when they go and review patients. (Doctor, Registrar in Palliative Medicine)*

In contrast, chaplains said they felt medication was not always the best solution for pain and being able to offer space to explore and discuss feelings, may itself improve discomfort. A phrase often used was space and time to “be with” patients.

*a patient themselves may have been erm I guess conditioned into thinking “I feel uncomfortable, I need a tablet “err in actual fact to have some time with somebody to express some of their anxieties and feelings that they had they may actually find well I don’t need that tablet any more. (Chaplain)*

Despite its apparent efficacy, “spending time” or informal psychological support was viewed as a “luxury” and not an essential role. Staff felt hospices were much busier than 10 years ago. Care was moving towards a task-orientated approach and staff were not consistently able to spend time as they wished.

P*2: You don’t always have the luxury… we’d always try but unfortunately I wouldn’t say we can always do it, especially not for half an hour. But If you can it’s nice isn’t it…(Nurse, inpatient unit).*

*P1: 10 years ago there would definitely be more time to spend talking to patients as opposed to now, yeah it just seems a heck of a lot busier (Nurse, inpatient unit).*

*P2: It can seem a bit more task orientated now which then you don’t know how then that is going to affect how the patients are feeling and then how that might manifest itself in their symptoms…. (Nurse, inpatient unit).*

## Discussion

### Main findings

We present necessary components of effective symptom management in palliative care as a simple, logical, data-derived model that considers the mixture and interdependence of psychological and physical needs, and interplay between patient, families and professionals. This model could be used to structure patient consultations to help evaluate a patient’s understanding, acceptance, expectations and goals and to help engage family with symptom management strategies.

Our data shows that although many medical approaches to symptom management target physical experience, it is impossible to consider these in isolation from psychospiritual, environmental and social aspects. Being treated as an inpatient within a palliative care service itself appeared to relieve patient’s symptoms before active intervention. This was attributed to “small things” such as a friendly welcome, feeling cared for and being informed about what was happening, factors also highlighted by www.hellomynameis.org.uk campaign. This raises the question as to whether integration of simple and non-expensive measures could improve effectiveness of symptom management and quality of palliative care non-hospice environments such as care homes and acute hospital wards.

The first step in our model is supporting and encouraging patients to engage with palliative care services. Forming an effective therapeutic partnership involved an interplay of managing expectations, maximising understanding and facilitating acceptance of limitations of treatment. An overarching theme was that staff found that a patient’s psychological distress was a barrier to forming this partnership and optimal engagement with therapeutic strategies. We described an example where non-medical intervention to assist a patient with family reconciliation, addressed the cause of this distress and facilitated more effect symptom relief.

Depression and anxiety are associated with worse symptom burden in advanced cancer patients and may impact on successful symptom management [14, 15]. Given the interplay between psychological distress and effective symptom management, early identification of psychological distress through routine screening is needed for patients with high symptom burden. Yet, most patients in palliative care are not routinely screened for psychological morbidity using a validated tool [16], meaning support may not always be targeted to all needs.

The distress thermometer, a simple one item tool, has been validated for screening for psychological morbidity within palliative care [17, 18]. In addition, the Integrated Palliative care Outcome Scale (IPOS) contains items for emotional concerns such as patient anxiety, depression, feeling at peace and communication issues and has been validated for use in advanced disease [19]. The routine use of these or similar tools may aid staff in identifying patients who have psychological distress which can be addressed by members of the standard care team and help identify those who may require a referral for specialist support such as recommended by National Institute for Health and Care Excellence (NICE) guidelines for improving supportive and palliative care for adults with cancer [20].

Hospice staff valued being able to “be with” patients and saw how this informal psychological support could lead to symptomatic relief. Yet due to increasing workload pressures necessitating a more task-orientated approach, they were not consistently able to do this. Just “being with” a patient has been shown to be an effective role for end of life volunteers in palliative care, whose input is linked to increased general wellbeing [21].

Our study highlighted support of and helping the family to engage was key in determining effectiveness of symptom management. A previous study examined nurses’ perception of barriers to effective symptom management in hospices in the USA and found family who were unable to implement or maintain treatment was a key barrier [22]. Empowering patients and family with understanding about symptoms and treatments was a facilitating factor. Fear of opioid addiction and syringe drivers were barriers to management of breathlessness and pain. Media messages about addiction as well as high profile investigations in recent history [23, 24], may have affected attitudes.

Decisions on how to proceed were team based. However, we found discordance between staff who felt their strength was working together but also reported a lack of understanding about the role of other specialities in their team. Unspoken rules about which aspects of care different categories of staff were responsible for could also be a barrier to effective team working. Improving communication within multi-disciplinary teams around services available and role boundaries might enable more effective symptom management.

Of three symptoms we specifically asked about (pain, breathlessness, fatigue), staff found it was most challenging to manage a patient’s fatigue effectively. Although fatigue troubled most patients, there was not a good evidence base upon which to form clinical decisions. There is a need for high-quality research into both pharmacological and non-pharmacological approaches to fatigue management.

### Strengths and limitations

Qualitative data derived from a multidisciplinary sample of staff of varying levels of experience over 5 independent hospice settings was used to inform development of a novel conceptual model of effective symptom management in palliative care. We gathered data from multidisciplinary staff who provide hospice-associated palliative care in the diverse settings of inpatients wards, outpatients and in the community. Interviews were conducted over a relatively short time frame using same procedures and research team. Participants were aware interviewers were University researchers rather than clinical staff, it was our intention this will have been conducive to obtaining a candid and accurate account of difficulties and facilitators associated with symptom management. It is also possible this discordance in interviewer-participant profession may have limited information divulged. Data was captured from staff employed by adult hospices. The knowledge gained from these specialists in symptom management might be transferable to other palliative care contexts such as hospital, primary care or paediatric patients but other context-specific barriers or facilitators are possible. Participants were questioned about their experience of managing pain, breathlessness and fatigue. If we had asked about different symptoms such as nausea, additional barriers and facilitators may have been identified. Participants were aware the focus of the research programme was advanced disease with particular emphasis on cancer patients (as the work was explicitly funded by a cancer research charity and this was clear in the participant information sheet). Participants, reflected upon their experience in managing symptoms, predominantly in cancer patients as the most common users of hospice service, but also in non-cancer patients. People with non-cancer conditions and different cancer types may experience a different symptom profile or trajectory which might have generated alternative data. We captured healthcare professional perspective of which factors influence effectiveness of their practice in symptom management. However, discrepancies in perception of symptom experience may differ between patients and staff or family. It is essential future research examines patient and family perceived barriers and facilitators to effective symptom management.

## Conclusions

We have broken down successful symptom management into logical steps of engagement, partnership, decision-making, and delivery. This novel and simple, data-derived model was derived from opinions and experience of diverse multidisciplinary team members providing care in outpatient, inpatient and community settings. We highlight how successful delivery of symptom management is facilitated by clinicians who enable engagement and partnership with patients and families, leading to shared decision-making.

The model has potential to enable improvement of symptom management by informing development of a consultation framework, treatment review or an aid to reflection on practice or service evaluation. The model could also be used to support behaviour change of staff to consider and act upon patient’s psychological distress at the earliest opportunity and consider the staff, family and patient partnership.

## Supplementary Information

**Additional file 1:** **Table 1.** Demographic information of participants.

**Additional file 2:** **Table 2.** Details of focus groups and interviews.

**Additional file 3:** **Table 3.** Illustrative extracts of data used to form the thematic framework.

**Additional file 4:** **Table 4.** Symptom management interventions identified though card sort exercise.

## Data Availability

The datasets generated and/or analysed during the current study are available from the corresponding author on reasonable request.
