# Supplementary material for: Conceptualising effective symptom management in palliative care: a novel model derived from qualitative data
Source: BMC Palliat Care. 2022 Feb 4;21:17. doi: 10.1186/s12904-022-00904-9 (PMC8815221; doi:10.1186/s12904-022-00904-9)
Supplement: Supplementary file 1 — Additional file 1: Table 1. Demographic information of participants. [file 12904_2022_904_MOESM1_ESM.docx]

| **Additional material Table 1.** Demographic information for participants in focus groups (n=53) and tailored interviews (n=8). Due to the gender imbalance in the palliative care workforce towards females, gender was not reported as this may compromise confidentiality. | | | | | | | | | | | |
| --- | --- | --- | --- | --- | --- | --- | --- | --- | --- | --- | --- |
| Age (years) | 18-24  1 | 25-34  11 | | | 35-44  16 | | 45-54  17 | | 55 +  7 | | No data  1 |
| Experience in palliative care (years) | Average 9.33 (range 6 weeks to 30 years) | | | | | | | | | | |
| Role | Nurse  20 | | | Doctor  11 | | | | Allied healthcare professional  22 | | | |
| Specialist palliative care qualification | 6 | | | 2 | | | | 1 | | | |
| Individual interview participants (n=8) | | | | | | | | | | | |
|  | 18-24  0 | | 25-34  4 | 35-44  0 | | 45-54  3 | | 55 +  1 | | No data  0 | |
| Years of experience in palliative care | Average 7.6 (range 0.20 to 20 years) | | | | | | | | | | |
| Role | Nurse  1 | | | Doctor  5 | | | | Allied healthcare professional  2 | | | |
| Specialist palliative care qualification | 0 | | | 1 | | | | 1 | | | |
