# Supplementary material for: Conceptualising effective symptom management in palliative care: a novel model derived from qualitative data
Source: BMC Palliat Care. 2022 Feb 4;21:17. doi: 10.1186/s12904-022-00904-9 (PMC8815221; doi:10.1186/s12904-022-00904-9)
Supplement: Supplementary file 2 — Additional file 2: Table 2. Details of focus groups and interviews. [file 12904_2022_904_MOESM2_ESM.docx]

| **Additional material Table 2.** Details of focus groups and interviews | | | | |
| --- | --- | --- | --- | --- |
|  | **Duration (minutes)** | **Location** | **Participants** | **Researchers** |
| Focus Group 1 | 68 | Hospice A | 3 x Allied healthcare professionals | EC, SP |
| Focus Group 2 | 63 | Hospice A | 4 x Nurses | EC, SP |
| Focus Group 3 | 62 | Hospice A | 3 x Doctors | EC, ZE |
| Focus Group 4 | 54 | Hospice B | 4 x Nurses | EC, SP |
| Focus Group 5 | 47 | Hospice B | 5 x Allied healthcare professionals | EC, ZE |
| Focus Group 6 | 60 | Hospice C | 3 x Doctors | EC, SP |
| Focus Group 7 | 46 | Hospice B | 2 x Doctors | EC |
| Focus Group 8 | 46 | Hospice C | 3 x Allied healthcare professionals | EC, SP |
| Focus Group 9 | 49 | Hospice C | 3 x Nurses | EC, ZE |
| Focus Group 10 | 53 | Hospice D | 4 x Allied healthcare professionals | EC, SP |
| Focus Group 11 | 46 | Hospice E | 5 x Nurses | EC |
| Focus Group 12 | 37 | Hospice D | 5 x Nurses | EC |
| Focus Group 13 | 40 | Hospice D | 3 x Doctors | EC |
| Focus Group 14 | 34 | Hospice E | 4 x Allied healthcare professionals | EC |
| Focus Group 15 | 42 | Hospice A | 4 x Allied healthcare professionals | EC |
| Interview 1 | 39 | Hospice B | Doctor | EC |
| Interview 2 | 43 | Hospice B | 2 x Doctors | EC |
| Interview 3 | 40 | Hospice E | Doctor | EC |
| Interview 4 | 46 | Hospice E | Allied healthcare professional | EC |
| Interview 5 | 40 | Hospice E | Allied healthcare professional | EC |
| Interview 6 | 31 | Hospice D | Doctor | EC |
| Interview 7 | 40 | Hospice A | Allied healthcare professional | EC |
