# Supplementary material for: Conceptualising effective symptom management in palliative care: a novel model derived from qualitative data
Source: BMC Palliat Care. 2022 Feb 4;21:17. doi: 10.1186/s12904-022-00904-9 (PMC8815221; doi:10.1186/s12904-022-00904-9)
Supplement: Supplementary file 3 — Additional file 3: Table 3. Illustrative extracts of data used to form the thematic framework. [file 12904_2022_904_MOESM3_ESM.docx]

**Supplementary Table 3**. Illustrative extracts of data used to form the thematic framework

| **What influences healthcare professional’s choice of approach?** | |
| --- | --- |
| **Guidelines and Evidence** | *I find fatigue really hard, and I do have a little bit of a heart-sink when patients say, “My main problem is fatigue,” because I feel like there’s nothing that I know of that really has good evidence behind it. (Registrar in Palliative Medicine)*  *It’s a big research hole, yeah I mean there is some evidence but not erm not, nothing that’s going to radically change our clinical practice which is what we need don’t we? We need a Cochrane review on fatigue with good studies in the review to guide us (Consultant in Palliative Medicine, in patient unit)*  *I don’t use weak opioids because the evidence is that they’re not as effective as appropriately used strong opioids and have more side effects than appropriately used strong opioids erm so I don’t use those because the pain ladder was a bunch of guys in the 1980’s who got together and thought this would be a good idea, it’s not actually evidence based at all erm so you can ignore it erm. Well you can absolutely ignore it because it’s based on nothing (Consultant in Palliative Medicine, in patient unit)* |
| **Experience** | *P3 We do that a lot, if somebody just says I’m feeling a bit uncomfortable it might then just lead into a conversation you spend half an hour with them and then actually you don’t want anything, they don’t need anything (Nurse, in patient)*  *P1 Especially if it’s been ward round and you know they’ve had some difficult discussions then you do notice that the pain can get worse and it’s just like having that time to talk to them about and reflect on what’s been said (Nurse, in patient unit)* |
| **Training** | *it’s just having that, making sure that everyone’s got that knowledge, not just the staff that have been here for years (Nurse, in patient unit)*  *And we’re trying to upskill on that you know we’re trying to increase our understanding of that but we’re not erm you know none of us are kind of CBT (Cognitive Behavioural Therapy) practitioners or anything like that or, and we haven’t had, we’re learning about ACT (Acceptance and Commitment Therapy) and that but we’re not trained in that so using principles of those things in what we’re doing with people so yeah (Physiotherapist).* |
| **Role definition and boundaries** | *P2 Then again that’s something we do very well here I think everyone’s very aware of their own personal limitations and there’s no kind of overstepping the mark really. You all know what you can say and if you’re not, as I say if you’ve any doubt just (Nurse, in patient unit)*  *P3 Keep quiet (Healthcare assistant, in patient unit)*  *P4 Don’t say anything (Healthcare assistant, in patient unit)* |
| **MDT decision making** | *so it’s having a multi-faceted approach to their pain. So it’s having a strong, a low dose strong opioid with non-opioid adjuvants with radiotherapy and imaging, with nerve blocks erm with, wish a, with good communication and with physical measures as well like TENS (Transcutaneous electrical nerve stimulation) or complimentary therapy or and you know it’s about addressing multi-faceted rather than putting all your eggs in one basket and looking at the patient as a holistic person and thinking what else, what other issues are going on here how am I going to manage this. (Consultant in Palliative Medicine, in patient unit).* |
| **Availability of services/staff** | *Yeah. It’s interesting, it’s a bit of a, at the moment it’s a bit of a GP lottery, postcode lottery about formal psychology support in sleep specifically (Doctor, GP trainee)*  *We now have a drop in psychologist who calls in every Friday afternoon (Nurse, ward manager)*  *psychologists the only thing I wish we had for longer ‘cos she’s only normally here for like a day isn’t she (Nurse, in patient unit)* |
| **Clinician-Patient relationship/rapport** | *I can think of a couple of patients where we have used that where they can’t, they can’t describe pain using words.  Some of them I’ve known draw or describe the colour or describe it as some sort of different entity and you have to unpick that…And it took us a while to work out that that was what he was actually trying to describe…she used to draw her pain and then we’d assess it on ward round and she’d have quite a different pain diary (Transfer of care sister, in patient unit)* |
| **Patient preferences** | *we quite often have patients in don’t we that have longstanding breathing problems and then they quite often got into quite bad habits of how they breathe or how they manage those episodes (Staff nurse, in patient unit)* |
| **Patient characteristics (including reversible causes)** | *people who have been drug users and addicts in the past often have unresolved pain, still need to work that out really ‘cos it’s really, really challenging. Tolerance they have for their own pain is just well (Ward manager/nurse)*  *‘Cos if they’re fairly straightforward, or even fairly complicated the community team will have dealt with it in community so the ones who come through and I think from a staff point of view of coping with it we probably need to be realistic that you know we’re not miracle workers. You know this is somebody who’s probably been through various stages of people trying to get their pain sorted elsewhere (Staff nurse, in patient unit)* |
| **Quality of life vs Treatment need** | *P1* *it just feels like more and more of our patients are having active aggressive treatment in their last week of life*  *I* *Yeah*  *P1* *And erm it’s really hard and I think it’s probably, it’s a fact of life and I don’t think it will change and I think we probably need more erm education for the nurses and healthcare assistants to appreciate why erm we have to treat reversible causes erm you know from I guess it’s a, you know a negligence point of view isn’t it (Nurse, in patient unit)*  *do I want to destroy my therapeutic relationship with this patient by doing what is technically correct or do I just want to accept the fact that this is how it is? and so I can do other work that’s going to help and maybe tackle it again down the line so everything we do is a trade-off of risk versus benefit because tactually you may say in a textbook sense removing the oxygen is the correct thing to do but if they then withdraw from palliative care services and then they start bouncing in and out of the acute trust well really you’ve probably done them more harm than good even though technically you were correct.(Doctor, Consultant in Palliative Medicine)* |
| **Staff time/burden** | *the challenge inevitably erm, the priority for the hospice is that the beds are filled and that people are receiving the care that they need. To the resources required, scheduling in time err for a nurse or whoever to have a specific amount of time you know within a framework err has cost implications and as much as it might be held up as an ideal, when push comes to shove, things that tend to go (Chaplain)*  *some people they have the pain relief and say no it’s not working but then they’ll say oh distraction helps and distraction’s great if you’ve got time to sit and talk to somebody. I’ve seen it once or twice with people but if you haven’t got the time to sit and talk (Healthcare Assistant, in patient unit)* |
| **What do HCP see as important factors in supporting delivery of effective care?** | |
| **Psychological support - Forma**l | *clinical psychology has been recognised as a scarce and targeted resource when there’s a particular source of the distress…if money was no object…we will probably say great well let’s get a few more clinical psychologists (Doctor, Consultant in Palliative Medicine)* |
| **Psychological support - Informal** | *psychologically distressed patients tend to be patients that you can do, make lots of changes pharmacologically but they never really feel like we’re on top of things necessarily or they become quite difficult to manage regarding kind of trying to change things (Doctor, Specialist Registrar)* |
| **Psychological support - For staff** | *there’s two patients stand out. They kind of make you wake up in a cold sweat and you’re thinking about them when you’re on call; you think, “Oh, if they call saying this patient’s reached their maximum on PRN (pro re nata) dosing and they’re still in loads of pain, I don’t know where to go from here”. It’s really difficult, and really quite emotionally draining. (Doctor, Registrar, inpatient Unit)*  *I think we have a really good, supportive team and manager and we have clinical supervision, that’s it, on a monthly basis. We have that one-to-one support.(Social worker)* |
| **Appropriate understanding, expectations, acceptance and goals Patients** | *every time they get into that panic of not being able to breathe that is going through the back of their mind that is this it? Is this the end this time it’s it, this time it’s got to be it. So I think psychologically for them that must be terrifying (Staff nurse, in patient unit)*  *I mean so it’s a bit of an old wives tale that people just go to the hospice to die, they actually don’t (Nurse, in patient unit)* |
| **Appropriate understanding, expectations, acceptance and goals - HCPs** | *I feel a bit guilty about it I mean we, our first instinct is to try and help people alleviate their pain, that’s what we want to do but when we can’t it gets very frustrating (Doctor, GP trainee)*  *P1 (Staff nurse, out patient unit) Recently on the news has been the whole debate about opiates and people becoming addicted and I think personally, more so over the last couple of years, people are much more reluctant to take things. They then have issues I think particularly now patients we see the GPs don’t give them enough supply of their medicines…*  *I Where do you think the error’s happened there, do you think it’s with the GP or ?*  *P5 (ward manager, in patient unit) Probably the GP*  *P4 (Staff nurse, out patient unit) I suppose you think they’re scared*  *P3 (staff nurse out patients unit) yeah*  *P4 (Staff nurse, out patient unit) Especially the Mr Shipman thing, they are the very people prescribing opiates* |
| **Appropriate understanding, expectations, acceptance and goals - Family** | *So often they, they work their family member up because they’re distressed so they sort of play off each other’s stress and the last thing you want when you’re breathless is to get more stressed and worked up and they sort of rely on, family members often push relatives to ask for interventions but actually the relative’s not asking ‘cos they know they don’t help…I think it’s very difficult to help with the pain like for a family member to help the situation is very difficult but again for them to make it worse is very easy.(Doctor, GP trainee)*  *Sometimes it’s the family I’m dealing with more than the patient (Healthcare Assistant, in patient unit)* |
| **Professional, service and referral factors**  **Continuity of care** | *I always feel it’s so important to get the patient at the right time. So sometimes I’d be planting the seed and then they would then ring back up a few months later and go, “I’m ready to listen now,” so I would then go back in and they’d be engaged at that point and wanting to make the changes to their routine and then sometimes they’d come through and they’d be ready and you would just work with them and go, “So what’s important to you?”.(Senior Occupational Therapist)* |
| **MDT working** | *within the multidisciplinary team and the erm clinical review meeting that we have each week all professionals are of an equal standing around the table as we review the patient’s needs it may be that we have the thoughts but it’s for the doctor or the nurse or some other to present that thought to the patient. Who presents the thought is immaterial the fact that they’re being looked at more holistically erm is the important thing.(Chaplain)* |
| **Palliative care philosophy and culture** | I *think they seriously thought that we plump pillows and hold people’s hands and say “there there”. We do not stop do we? We are multi skilled, I’m dead proud of what we do.”(Nurse, inpatient unit)* |
| **Physical environment and facilities** | *I couldn’t imagine doing what I do in a hospital, it would finish me off completely. Not least because there’s like 400 miles worth of corridors in hospital. But no it’s a completely different environment. It’s one of the main feedbacks that we get from patients who come here from hospital you know you can visibly see their shoulders lowering again as they settle into the hospice. (Social worker)*  *it’s nice to have the gardens here that are wheelchair accessible because erm people will put their relative into the wheelchair, take them out into the garden and they just get that sense of being outside... it’s not uncommon for families to put somebody into a wheelchair and take them down to the pub and it just adds a bit more, bit more normality and nice to see someone with your family having a drink.(Doctor, Consultant in Palliative Medicine)* |
| **Referral process and delays** | *You have to try and top trumps, who’s got what but we are quite good at sitting down, I mean I’ve just had it today where we’ve had 3 people wanting a referral in and we’ve had to bounce one away saying no but this person and this person yeah they can come in but even though you phoned first no. Because they can be managed at home a bit better whereas these 2 people they need, they clearly need to come in (Nurse, community palliative care)*  *If I get them in earlier, during their illness, they can take advantage of more of the things that we can offer and they’re actually, most people are actually quite surprised when they come, I expected it to be a dark scary place and it’s not so we’re now starting to do assessment appointments so when people are referred in, we phone them and they come in, talk to us, have a look round erm see what we can offer them and what they need really (Nurse, out patient unit)*  *I tried to get to refer a few patients, I was disappointed that I couldn’t get some patients to be seen or have appointments for them within the time frame that I hoped to achieve. Because if you wait three weeks, then you’re unlikely to keep the patients for three weeks to manage pain; you would maybe aim to do things within a week or two. So, you don’t see results from the Pain Team.(Speciality doctor in Palliative Medicine)* |
